# Can the Brain Build Probability Distributions?

**DOI:** 10.3389/fpsyg.2021.596231

**Published:** 2021-03-25

**Authors:** Marcus Lindskog, Pär Nyström, Gustaf Gredebäck

**Affiliations:** ^1^Department of Psychology, Uppsala University, Uppsala, Sweden; ^2^Department of Education, Uppsala University, Uppsala, Sweden

**Keywords:** probabilistic mind, EEG, probability distribution, neural likelihood response, experienced data

## Abstract

How humans efficiently operate in a world with massive amounts of data that need to be processed, stored, and recalled has long been an unsettled question. Our physical and social environment needs to be represented in a structured way, which could be achieved by reducing input to latent variables in the form of probability distributions, as proposed by influential, probabilistic accounts of cognition and perception. However, few studies have investigated the neural processes underlying the brain’s potential ability to represent a probability distribution’s complex, global features. Here, we presented participants with a sequence of tones that formed a normal or a bimodal distribution. Using a novel, single-trial EEG analysis, we demonstrate a neural response that indexes the likelihood of an item, given previously presented items, and corresponds to the experienced tones’ distribution. Our results indicate that the adult human brain can build a representation of the complex, global pattern of a probability distribution and offer a novel tool for an in-depth understanding of related neural mechanics.

## Introduction

According to recent accounts of cognition (Chater et al., 2006) and perception (Knill and Pouget, 2004; Yuille and Kersten, 2006), the human mind is probabilistic, in that it represents data in the form of probability distributions (Knill and Pouget, 2004; Clark, 2013; Ma and Jazayeri, 2014). A probabilistic mind does not have to represent every feature of every piece of data it encounters; instead, data can be summarized with only a few parameters that capture its distribution (such as the central tendency, variance, and the approximate number of observations). According to these accounts, knowledge about the world is acquired through an inferential process (Ma et al., 2006; Fiser et al., 2010; Tenenbaum et al., 2011; Pouget et al., 2013; Ma and Jazayeri, 2014) over probability distributions (Knill and Pouget, 2004; Clark, 2013), where existing knowledge (prior distributions) is updated in the light of new data.

Evidence in support of a probabilistic mind comes both from studies investigating cognitive processes such as language acquisition (Kalish et al., 2007), memory (Anderson and Milson, 1989), intuitive physics (Téglás et al., 2011), and intuitive statistics (Griffiths and Tenenbaum, 2006; Gweon et al., 2010; Griffiths et al., 2011) as well as from studies investigating perceptual processes such as visual perception (Ma et al., 2006; Yuille and Kersten, 2006; Pouget et al., 2013), auditory perception (Pearce et al., 2010; Winkler and Czigler, 2012; Frost et al., 2016), sensorimotor learning (Körding and Wolpert, 2004), and statistical learning (Conway, 2020). In both of these domains, probabilistic computational models can account for a diverse set of human behavior and perceptual processes. Probabilistic computational models have also provided a proof-of-concept that probability distributions could be implemented in groups of neurons (Ma et al., 2006; Fiser et al., 2010, p. 201; Pouget et al., 2013; Ma and Jazayeri, 2014).

Furthermore, studies investigating statistical learning (sometimes referred to as implicit learning or sequence learning) have shown that people can learn probabilistic patterns in sequentially presented data (Saffran et al., 1996; Fiser and Aslin, 2002; Kirkham et al., 2002; Fiser et al., 2010) and that specific brain regions are engaged during such learning processes (Dehaene et al., 2015; Conway, 2020). However, most studies investigating the neural representation of statistical learning have focused on local probabilistic features of the presented data, such as associations between adjacent items or adjacent chunks. In contrast, much less attention has been given to complex, global patterns, such as distributional features, that require the integration of information over longer time-scales (Dehaene et al., 2015; Conway, 2020).

Taken together, findings from the previous literature strongly suggest that the brain can represent even the complex, global patterns of a probability distribution on the neural level and use the representations for perceptual and cognitive tasks. However, although many prominent theories in cognitive science rely on the assumption that the brain builds probability distributions, it has been argued that probabilistic representations might be too complex and far beyond the computational ability of the human brain (Mozer et al., 2008; Jones and Love, 2011; Bowers and Davis, 2012; Marcus and Davis, 2013, p. 20). This critique is warranted, given that little previous research has provided neurophysiological evidence that the brain can represent the complex, global patterns of probability distributions, as conjectured by the probabilistic accounts of the mind.

Here, we investigated whether the brain can build probability distributions to represent experienced data and use this global representation to evaluate the likelihood of new information. We reasoned that three pieces of neural evidence were required for concluding that the brain builds such representations; indexing, correspondence, and flexibility. First, the neural response should *index* the likelihood of a single presented item, given previously presented items. Previous research has shown a distinct neural response to unexpected events (Winkler, 2007). However, our indexing criterion requires, in addition to a response to unexpected events, that the response scales to the degree of unexpectedness. Significantly, this response should not be driven by potential covariates, such as how often an item is presented or its similarity to other items. Second, the neural response as a function of item likelihood should *correspond* to the distribution of the presented data, such that the neural response approximates the underlying structure of the data. Put differently; a normally distributed dataset should result in a neural response that approximately follows a normal (gaussian) curve. Previous studies indicate that people make use of mental shortcuts (heuristics) when dealing with uncertainty (Gigerenzer and Todd, 2001; Gilovich et al., 2002) and try to reduce experienced stimuli to simple parameters such as range and frequency (Parducci, 1965). Indeed, there is even evidence that the brain considers small variations within a range when determining if a new stimulus is unexpected (Winkler et al., 1990). Finding evidence for *indexing* and *correspondence* would indicate that the brain represents a probability distribution, rather than using a shortcut or simple parameter. Finally, the neural response should be *flexible* in that it should vary with the structure of the presented data. This feature is important because probabilistic accounts of cognition and perception suggest that existing representations are fine-tuned as new information become available (Knill and Pouget, 2004; Ma et al., 2006; Fiser et al., 2010; Tenenbaum et al., 2011; Winkler and Czigler, 2012; Clark, 2013; Pouget et al., 2013; Ma and Jazayeri, 2014). Thus, if a presented dataset was bimodal instead of normally distributed, the neural response should also have two peaks.

**FIGURE 1:**
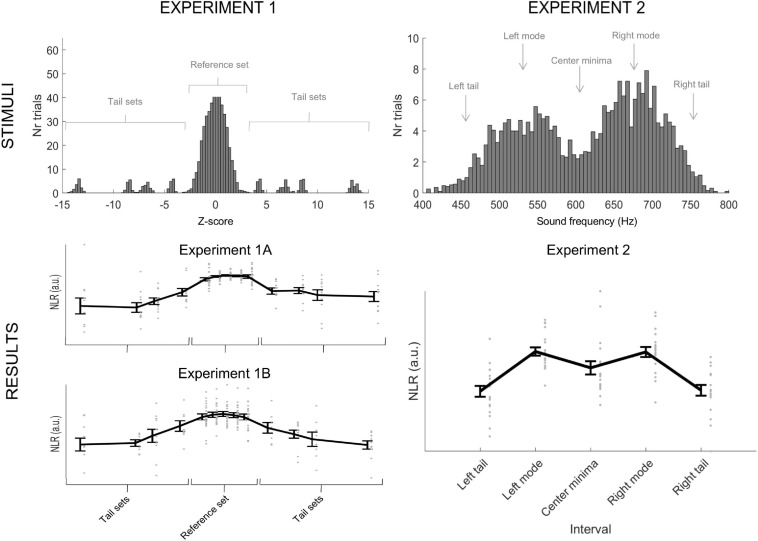
Illustration of stimuli and results for Experiments 1A, 1B, and 2, respectively. **(Top)** Histogram of the auditory stimuli presented to participants in Experiments 1 (1A and 1B) and 2, respectively. In Experiment 1A and 1B, we presented a total of 620 tones, 500 tones from a reference set, and 120 tones from four tail sets (30 from each distribution). Thus, tones from the tail sets were equally frequent. The histogram illustrates stimuli collapsed over all references sets used and their corresponding tail sets. In Experiment 2, we presented a total of 500 tones that followed a bimodal distribution. The figure includes an illustration of the two tails’ position, modes, and the center minima used to analyze the data from Experiment 2. **(Bottom)** Individual data points from Experiment 1A, Experiment 1B, and Experiment 2, respectively, with the mean and standard error (whiskers). The figure depicts the negative normalized NLR. In Experiment 1A and 1B, the individuals’ responses resemble a normal curve, and the response to the tail sets is not categorically flat. In Experiment 2, the brain responses flexibly mirror the presented stimuli and not a normal curve.

**FIGURE 2:**
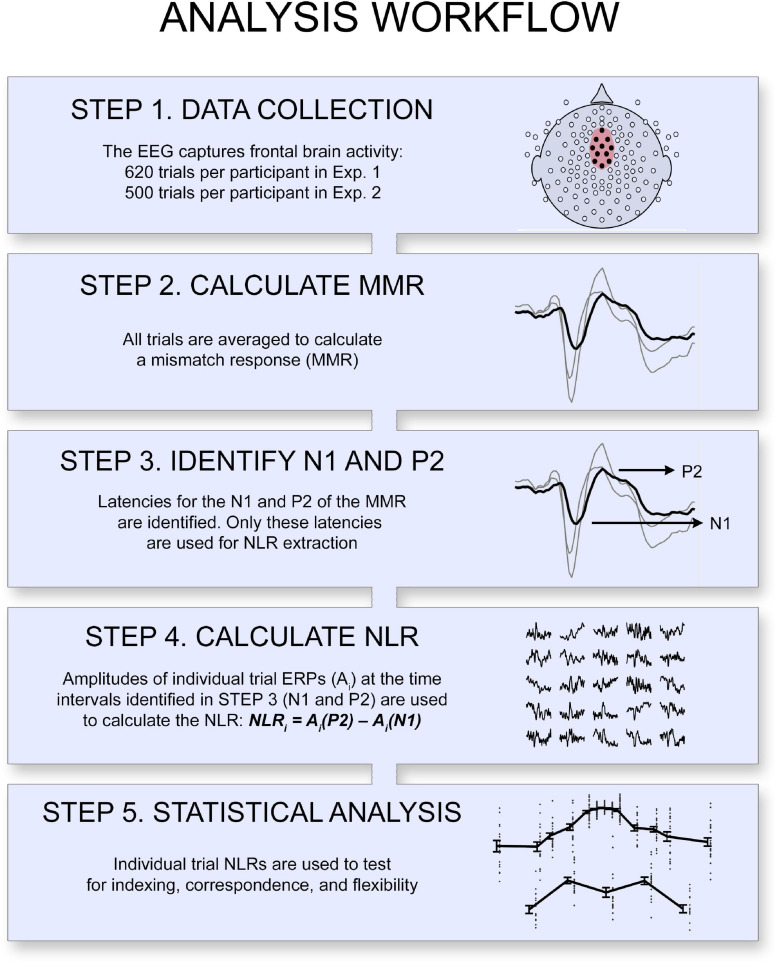
Overview of the analysis workflow. A neural likelihood response (NLR) was calculated for each item as the single-trial ERP amplitude difference at the latencies of the mismatch response’s positive peak (P2_MMR_) and negative peak (N1_MMR_). The statistical analyses tested the indexing-, correspondence-, and flexibility properties of the NLR.

In the current study, we combine insights from previous work investigating how the brain responds to unexpected auditory events (Kujala et al., 2007; Winkler, 2007; Winkler and Czigler, 2012) with those investigating how the brain learns environmental structure (Dehaene et al., 2015; Conway, 2020). However, we move beyond such studies by focusing on the neural responses associated with the probability distribution’s complex, global patterns. We measured electric brain responses elicited while participants passively listened to a sequence of tones, the frequencies of which followed an approximately normal distribution (Experiments 1A and 1B, see Figure 1) or a bimodal distribution (Experiment 2., see Figure 1). In the main text, we focus on the functional response due to the theoretical implications and provide information on the topographical response in the Supplementary Information (SI). We were interested in single-trial activation, as we wished to evaluate whether the brain responds to the likelihood of each tone, which cannot be achieved with traditional measures of event-related potential (grand average ERPs). Therefore, we devised a new analysis method and calculated a measure, the Neural Likelihood Response (NLR), of the brain’s response to each different tone (see Figure 2 and section “Materials and Methods” for details). The NLR was calculated as the difference in amplitude within trials at the latencies of the mismatch response’s (MMR) most prominent positive (P2_MMR_) peak and negative peak (N1_MMR_) at a frontal region of the scalp (Figure 2, and see the section “Materials and Methods” for detailed information on how the P2_MMR_ and N1_MMR_ were identified). The amplitude difference, rather than absolute voltage, was used to reduce inter-trial variability due to individual EEG trials often containing drift and noise. We argued that if the brain builds a probability distribution of the experienced data, the NLR should exhibit indexing, correspondence, and flexibility, as outlined above.

## Experiment 1A

### Methods

#### Participants

Participants were 22 university students (13 women, mean ± SD age 26.5 ± 9.3, range 19–63 years) recruited through flyers on the local campus. We collected data from two additional participants, who were not included in the final analysis. One was not included due to technical problems and one was not included because a mismatch negativity response could not be detected due to issues with impedance or too many movement artifacts. Participants received a cinema voucher (worth ∼10 EUR) or course credits for participating.

#### Ethics

The study was approved by the local ethical committee (Regionala Etikprövningsnämnden) and was performed following the Helsinki Declaration of 1964. Before starting the EEG recordings, all participants were familiarized with the lab, and the application of the EEG was described. Participation was voluntary, and it was possible to quit the experiment at any time. However, no one stopped the experiment early. Informed written consent was obtained from all participants.

#### Procedure

Before the EEG recording, all participants’ hearing was tested by creating audiograms based on perceived volume at different frequencies relative to a reference tone using pure sine waves as in the actual experiment. This procedure took approximately 8–10 min and screened the participant for hearing impairments. Normal hearing was requested during recruitment, and no participants were excluded because of their audiograms. Participants were recorded while sitting relaxed in a comfortable chair. Participants could sit with open or closed eyes but were instructed to move their eyes and blink as little as possible.

#### Stimuli

Stimuli consisted of pure sine wave tones with a duration of 100 ms and a random inter-stimuli interval of 800–1,100 ms. We presented 620 tones, which included four tail sets interleaved in a larger reference set. The tones were presented in a continuous sequence that lasted approximately 12 min. Tones in the *reference set* (500) were drawn from one of four normal distributions; two narrow distributions [*s* = 30: *N*(600, 30), *N*(700, 30)] and two broad distributions [*s* = 60: *N*(600, 60), or *N*(700, 60)]. We manipulated which set participants were presented with between-subjects by cycling through the four possible combinations throughout both Experiments 1A and 1B (*n* = 10, 10, 12, and 9 for each combination, respectively).

**FIGURE 3:**
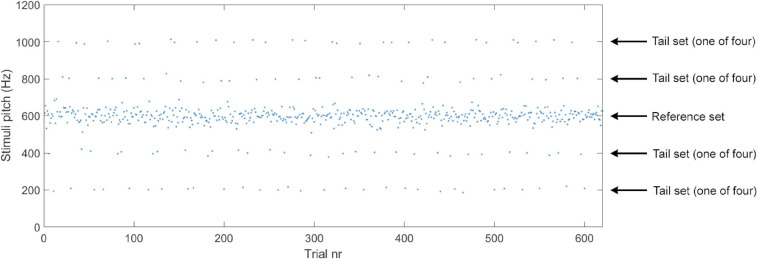
Example of stimuli presentation in Experiments 1A and 1B with pitch as a function of trial. See main text for details regarding the properties of the reference set and tail sets.

Tones for the *tail sets* were drawn from four normal distributions (30 tones from each) with a standard deviation of 10 Hz and means ±200 Hz and ±400 Hz (i.e., on both sides of the reference set) that of the reference distribution (see Figure 1 for an illustration of the reference and tail sets). We included both positive and negative deviations in the tail sets to control for the effects of absolute pitch. The tail tones were always presented with 4–6 reference tones in between and blocked so that all four tail distributions contributed with one trial (in random order) before being presented again (see Figure 3). No other order or blocking rules were used. Importantly, the four tail distributions contributed an equal number of tones (*n* = 30) and were equally frequent.

Suppose the mind treats the tones inside and outside the reference set as categorically different. In that case, we expect the tones in the tail set to be outside of the category boundaries of the reference set and give rise to a categorically different neural response (Winkler et al., 1990). In contrast, if the brain represents the probabilistic information in the experienced data, we expect the neural response to the tones in the reference and tail sets to be on a continuum. To allow for the possibility of categorically different responses to the reference and tail sets, these two cannot overlap. Therefore, the reference sets with *s* = 60 were truncated at ±100 Hz from the mean. This procedure resulted in an empirical standard deviation (SD) of 48 in these sets. For participants that experienced a narrow reference set (*s* = 30), the tail sets had means corresponding to 6.7*s* and 13.3*s*, while for participants that experienced a wide reference set (*s* = 48), the tail sets had means corresponding to 4.2*s* and 8.3*s*. Thus, across all participants, eight tail sets were used.

#### EEG-Analysis and NRL Extraction

Geodesic Sensor Nets with 128 channels (HCGSN 130; EGI, Eugene, OR, United States) were used to record EEG signals with a sample rate of 250 Hz. The signal relative to the vertex reference was amplified by an EGI Net amplifier (GES 300 Amp; EGI, Eugene, OR, United States) and stored for offline analysis. The data were then digitally filtered (0.3–30 Hz) and segmented into trials from 300 ms before the sound onset to 1,000 ms after. Trials were baseline corrected by subtracting the mean of the pre-sound interval (−300 to 0 ms). The data were then resampled to 100 Hz to reduce memory load and re-referenced to an average reference.

A region of interest (ROI) containing 11 channels [EGI channels 5, 6, 7, 11 (Fz), 12, 13, 31, 55, 80, 106, 112] was defined based on previous literature (Kujala et al., 2007, who suggested the Fz and frontal channels) and on visual inspection of the grand average ERPs (which suggested that the overall EEG response for all pitches together extended over the central electrodes; note that these channel locations are in line with studies reviewed in Näätänen et al., 2007). The most frontal electrode (11) corresponds to the Fz channel in the 10–20-system, and the channels 7, 31, 55, 80, and 106 surrounded the reference at the Cz channel location (see Figure 2 and Supplementary Figures 1, 4, 5, 7 for visual guidance). The ROI thus covered both frontal and central areas typically included when the central auditory function is measured (Näätänen et al., 2007). These channels were averaged, and a mismatch response (MMR) was calculated from the ERP average of tail trials (deviant trials) minus reference trials (frequent trials) (Kujala et al., 2007; Winkler, 2007). To exclude artifacts originating from eye and body movement and eye blinks, trials with an amplitude range exceeding 100 μV were excluded in individual channels before averaging. Individuals’ time points for the mismatch response’s most prominent negative (N1_MMR_) and positive peaks (P2_MMR_) were identified by a semi-automatic procedure, using visual inspection and manually marking the MMR waveform, where the position automatically snapped to the local minima or maxima. The identified time points were then used to calculate an EEG amplitude difference in all trials (i.e., within single trial ERPs), to capture the brain response to individual tones (see Figure 2 for an illustration). Because our aim was to assess brain activity to individual tones, we did not use the traditional mismatch negativity (MMN) component (Kujala et al., 2007; Näätänen et al., 2007), which is calculated from averaged responses of multiple trials. The MMN is a very stable EEG component that has been shown to measure central auditory function and responds to deviations in auditory stimuli structure (Kujala et al., 2007; Näätänen et al., 2007; Winkler, 2007). At a single-trial level, the ERP components are rarely visible, as they are very small compared to environmental noise and low-frequency drifts in the EEG signal. However, a difference measure between two points close in time is relatively robust to such low-frequency noise. Accordingly, we used this approach to reduce inter-trial variability. We here assume that the N1_MMR_ − P2_MMR_ difference represents a neural likelihood response (NLR), which is intended to be a single-trial equivalent to the MMR. We used the NLR as the dependent measure in all subsequent analyses. It is important to note that the two peaks (N1_MMR_ and P2_MMR_) were identified based on the mismatch response, a measure based on averages. The NLR was calculated at single-trial ERPs after these peaks had been identified. Participants with poor signal-to-noise ratio, without clear MMR components, were excluded from further analysis (*n* = 8, Experiments 1A and 1B together). The mean latency (of Experiments 1A and 1B together) of N1_MMR_ was 131 ms (*SD* = 23, range = 90–180), and *M* = 221 ms (*SD* = 25, range = 150–250) for P2_MMR_.

#### Model Fitting

Model fitting was performed using the MATLAB “fit” command in the curve fitting toolbox, using the Trust-Region algorithm and a non-linear least-squares fitting method with a maximum of 1,000,000 iterations and evaluations of the model. We used frequency *z*-scores as the *x*-data and the NLR as the *y*-data and averaged all data points within one (1) *z* intervals centered on *z*-positions that captured all tail distributions and split the reference distribution into thirteen bins (i.e., the *z* bins were −13.63, −8.375, −6.75, −4.125, −2, −1, 0, 1, 2, 4.24, 6.75, 8.5, 13.75). All individuals’ data points used for modeling are illustrated as gray dots in Figure 1. The normal distribution was fitted individually for each participant using *NLR* = *a* + *normpdf* (*z*, *b*, *c*) ⋅ *d*, where NLR is the negative normalized neural likelihood response, *z* is the *z*-position, and *a, b, c*, and *d* are free parameters. In contrast, the square distribution was fitted using *NLR* = *a* + *c*/(1 + *exp*((*abs*(*z* − *e*) − *d*) ⋅ *b*)), where *a, b, c, d*, and *e* are free parameters. Thus, the square function was not a pure square but a continuous approximation to meet the fitting procedure’s requirements. The difference between adjusted *r*^2^ values for each participant’s normal and square distributions was tested with a paired *t*-test.

### Results and Discussion – Experiment 1A

We first asked if the NLR *indexes* the likelihood of individual items in a distribution. With the help of a linear mixed model [NLR ∼ 1 + Abs(*z*) + (1| ID), in Wilkinson notation] using all individual trial NLRs in the reference set as dependent variable and with absolute *z*-score of the presented frequencies as a fixed factor grouped within-subjects (random intercept), we demonstrate a significant effect [*F*(1,8430) = 4.09, *p* = 0.043]^1^ of the absolute *z*-score (*b* = 0.28, 95% CI = [0.008, 0.556], *p* = 0.043). Table 1 summarizes the fixed-effects parameter estimates of the model. In other words, the brain response increased as the likelihood decreased, which is supportive of a probabilistic account of the mind.

**TABLE 1:** Fixed effects parameter estimates for model using reference set.

			95% Confidence Interval				
Names	Estimate	*SE*	Lower	Upper	df	*t*	*p*
(Intercept)	0.342	0.328	−0.30124	0.986	18.1	1.04	0.311
Abs(*z*)	0.282	0.140	0.00874	0.556	8429.9	2.02	0.043

Next, we wanted to ascertain that the effect above was not driven by how often an item is presented. Put differently, it is possible that the NLR is influenced by the larger number of trials presented close to the reference set’s mean. If so, the NLR would index how often an item is presented, rather than how likely an item is. To address this possibility, we evaluated if the brain response varied as a function of likelihood even when holding item occurrence constant using a linear mixed model on all individual trial NLRs in the tail sets, with absolute *z*-score as a fixed factor grouped within-subjects (random intercept). Note that the frequency of the tones is identical for the tail sets, but they differ in terms of likelihood. Analyzing the tail sets also showed a significant effect [*F*(1,1258) = 18.6, *p* < 0.001] of the absolute z-score (*b* = 0.24, 95% CI = [0.13, 0.37]). Table 2 summarizes the fixed-effects parameter estimates of the model. Thus, not only does the brain response increase as the likelihood decrease, it does so even when keeping the frequency of the tones constant (see Figure 1).

**TABLE 2:** Fixed effects parameter estimates for model using tail sets.

			95% Confidence Interval				
Names	Estimate	*SE*	Lower	Upper	df	*t*	*p*
I(ntercept)	3.234	0.3737	2.502	3.967	16.5	8.65	<0.001
Abs(z)	0.244	0.0567	0.133	0.356	1257.6	4.31	<0.001

It is possible that the exhibited scaling of the NLR could arise due to some process other than the brain building a probability distribution to represent experienced data. Although the NLR indexes the likelihood of individual items, there might not be a correspondence between the NLR as a function of likelihood and the structure of the presented data. Previous research using auditory stimuli has indicated that when a deviant sound follows a sequence of repetitive standard tones (i.e., in “oddball” paradigms), a mismatch negativity (MMN) event-related brain potential (Kujala et al., 2007; Winkler, 2007) can be observed. The MMN is elicited regardless of whether participants are engaged with or ignores the sounds. Thus, it is possible that the observed scaling of the NLR is a result of a process that treats all of the tones in the reference distribution as standards and the tones in the tail distributions as oddballs. Previous research has indicated that even when small variations are introduced to the standard tone, an MMN is elicited by tones outside of the standard tone’s range (Winkler et al., 1990). A further alternative process, albeit related, is that the brain structures the experienced data into categories (Ashby and Maddox, 2005). Humans form categories from an early age (Mareschal and Quinn, 2001), and categories are essential data management tools throughout life (Ashby and Maddox, 2005). There is also clear neural evidence that the brain can create categories (Grinband et al., 2006). There have been several suggestions regarding what information people use to form perceptual categories (Ashby and Maddox, 2005). One possibility is that people rely on the range and frequency of the presented stimuli, as suggested by range-frequency-theory (Parducci, 1965). As suggested by the generalized context model, another option is that people store individual items in memory and categorize new items based on their similarity to the previously stored items (Nosofsky, 1986, 1988). However, regardless of the exact mechanism, a categorization process will divide the range of tones into separated category regions.

**FIGURE 4:**
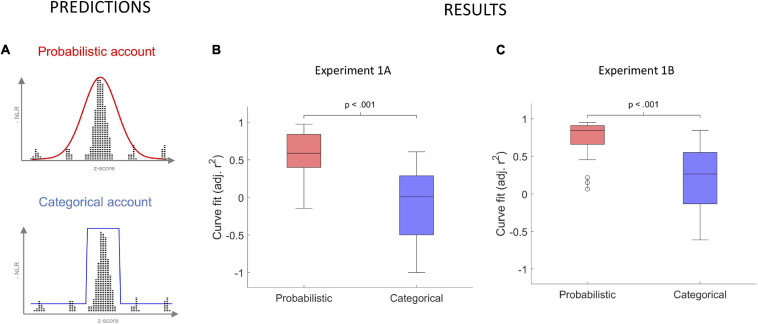
Predictions from the probabilistic and categorical account together with results showing the correspondence between the functional form of the NLR and the distribution of the data. **(A)** The red and blue curves illustrate predicted negative NLR for the probabilistic and categorical accounts, respectively. **(B)** Box plot of individual adjusted r^2^ for the fit of the normal (probabilistic) and square curves (categorical) in Experiment 1A. The normal curve provided a significantly better fit than the square (paired *t*-test, *p* < 0.001). **(C)** Box plot of individual adjusted *r*^2^ for the fit of the normal (probabilistic) and square curves (categorical) in Experiment 1B. The normal curve provided a significantly better fit than the square (paired *t*-test, *p* < 0.001).

Thus, both the MMN and categorization accounts will treat items within and outside the reference distribution as categorically different. Hence, we will refer to these two possibilities as the *categorical* account. It should be noted that there is evidence suggesting that the amplitude of the MMN can capture the degree of deviance from the standard tone (Sams et al., 1985; Frost et al., 2016), and it has been suggested that the MMN is an integral part of a predictive coding system in the brain (Winkler and Czigler, 2012). However, it is still unclear if the MMN can capture the full complexity of a probability distribution. The categorical account predicts the NLR to be the same or similar for all items within the reference distribution but different for all items outside. In terms of a function, such a pattern should be well approximated by a square function. Figure 4A illustrates the diverging predictions made from the probabilistic and categorical accounts, respectively.

We investigated if there is a correspondence between the NLR and the distribution of the data, such that the neural response approximates a normal (gaussian) curve. We evaluated the fit in terms of the adjusted *r*^2^ of a normal and square curve for each participants’ negative normalized NLRs^2^. We used transformed, rather than raw, NLRs to ensure that standard normal and square curves could be fitted to the data. For more information about the model fitting procedure, see the Methods. Overall, the normal curve provided a better fit to the data than the square. As illustrated in Figure 4B, the difference in adjusted *r*^2^, *M*_norm_ = 0.57, *SD*_norm_ = 0.28, *M*_square_ = 0.10, *SD*_square_ = 0.51, was significant, *t*(21) = 8.1, *p* < 0.001, Cohens’ *d* = 1.72. Although the normal curve had fewer model parameters, the unadjusted *r*^2^ was also significantly higher, *t*(21) = 6.8, *p* < 0.001, Cohen’s *d* = 1.46, for the normal curve, *M*_norm_ = 0.73, *SD*_norm_ = 0.18, than for the square curve, *M*_square_ = 0.45, *SD*_square_ = 0.26. These data show that the brain builds probability distributions because the neural response resemble a normal curve more than a square.

## Experiment 1B

Because our analysis method is new, we collected a second sample of participants in an effort replicated the findings of Experiment 1A using the same methods and the same analyses approach.

### Methods

#### Participants

Participants were 19 university students (11 women, mean ± SD age 26.1 ± 5.45, range 20–39 years) recruited through flyers on the local campus. We collected data from seven additional participants who were not included in the final analysis because a mismatch negativity response could not be detected due to issues with impedance or too many movement artifacts.

#### Procedure, Stimuli, and Analyses

Participants were recorded as in Experiment 1A. We used the same stimuli and analyses as in Experiment 1A. Participants in Experiment 1B also completed a questionnaire assessing sensory processing sensitivity after the EEG session. Analysis of this questionnaire is outside the scope of the current study and will be reported elsewhere.

### Results and Discussion – Experiment 1B

As in Experiment 1A, we first investigated if the asked if the NLR *indexes* the likelihood of individual items in a distribution. Using the same analyses approach, we found a trend in the same direction (*b* = 0.19, 95% CI = [−0.027, 0.41], *p* = 0.09), that did not reach conventional levels of statistical significance effect [*F*(1,10558) = 2.95, *p* = 0.086]. Table 3 summarizes the fixed-effects parameter estimates of the model.

**TABLE 3:** Fixed effects parameter estimates for model using reference set.

			95% Confidence Interval				
Names	Estimate	*SE*	Lower	Upper	df	*t*	*p*
(Intercept)	0.256	0.233	−0.2014	0.713	21.0	1.10	0.285
Abs(*z*)	0.193	0.112	−0.0272	0.414	10557.9	1.72	0.086

However, directly replicating the results of Experiment 1B, we found a significant effect, *F*(1,985) = 9.49, *p* < 0.001, of the absolute *z*-score, *b* = 0.14, 95% CI = [0.05, 0.23], when analyzing the tail sets. Table 4 summarizes the fixed-effects parameter estimates of the model.

**TABLE 4:** Fixed effects parameter estimates for model using tail sets.

			95% Confidence Interval				
Names	Estimate	*SE*	Lower	Upper	df	*t*	*p*
(Intercept)	2.215	0.2449	1.7346	2.694	20.8	9.04	<0.001
Abs(*z*)	0.139	0.0451	0.0506	0.227	984.8	3.08	0.002

Finally, as in Experiment 1A, we evaluated the fit of the NLR to a normal and a square curve to investigate if it exhibited correspondence. Replicating the findings form Experiment 1A, the results, summarized in Figure 4C, indicated an overall better fit, *t*(18) = 6.6, *p* < 0.001, Cohens’ *d* = 1.52, in terms of adjusted *r*^2^, for the normal, *M*_norm_ = 0.70, *SD*_norm_ = 0.28, than for the square, *M*_square_ = 0.19, *SD*_square_ = 0.46, curve. Furthermore, as in Experiment 1A, the unadjusted *r*^2^ was also significantly higher *t*(18) = 5.6, *p* < 0.001, Cohen’s *d* = 1.29, for the normal curve, *M*_norm_ = 0.81, *SD*_norm_ = 0.18, than for the square curve, *M*_square_ = 0.60, *SD*_square_ = 0.23.

Over two experiments, we found very similar results. For indexing, the analyses of the tail sets showed the same statistically significant result in both experiments. Although the direction of the effect for the analysis of the references was the same in both experiments, it did not reach conventional levels of statistical significance in Experiment 1B. Finally, for the correspondence analysis, the normal curve provided better fit to the data than the square curve in both experiments.

### Discussion – Experiments 1A and 1B

Taken together, Experiments 1A and 1B indicated that the NLR exhibits both indexing and correspondence. However, one final piece of neural evidence is needed to support the idea of a probabilistic mind. The neural response needs to flexibly change with the presented data’s distribution, demonstrating sensitivity to the environment (Knill and Pouget, 2004; Ma et al., 2006; Fiser et al., 2010; Conway, 2020). Also, we need to rule out one potential alternative explanation of the previous results. Namely, the brain relies on similarity (Nosofsky, 1988; Ashby and Maddox, 2005) rather than likelihood when processing the presented data. Experiments 1A and 1B cannot provide this crucial piece of evidence because we only present participants with a single distribution, the normal distribution, in which likelihood and similarity are confounded.

## Experiment 2

In Experiment 2, to address this, participants were presented with a sequence of 500 tones following a bimodal distribution (Figure 1, see section “Materials and Methods” for details on how the frequency distributions were created). To analyze the data, we divided the range of the pitch into five intervals for the two tails, the two modes, and the center minima of the distribution, respectively, and analyzed, for each participant, the NLR of the 50 tones closest to these positions, giving a total of 250 analyzed trials per subject. Choosing the 50 closest points was an arbitrary choice, which balanced the number of trials needed to get a reliable ERP for the MMR with the need to have a separation between the intervals. Suppose the brain builds a representation that flexibly changes with the presented data’s structure and uses likelihood as the representation metric, rather than similarity. In that case, it should be reflected by a negative normalized NLR with two peaks over the five intervals.

### Methods

#### Participants

Participants were 19 university students (11 women, mean ± SD age 33.8 ± 4.1, range 26–41 years) recruited through flyers on the local campus. We collected data from an additional 13 participants who were not included in the final analysis. Three participants were excluded due to technical problems with the stimuli presentation, and 10 participants were excluded due to a broken reference channel on one of the EEG nets, leading to noisy data that prevented identification of N1 and P2. Participants received a cinema voucher (worth ∼10 EUR) or course credits for participating.

#### Ethics

As in Experiments 1A and 1B, the study was approved by the local ethical committee (Regionala Etikprövningsnämnden) and was performed following the Helsinki Declaration of 1964. Before starting the EEG recordings, all participants were familiarized with the lab, and the application of the EEG was described. Participation was voluntary, and it was possible to quit the experiment at any time. However, no one stopped the experiment early. Informed written consent was obtained from all participants.

#### Procedure

Participants were recorded as in Experiments 1A and 1B.

#### Stimuli

As in Experiments 1A and 1B, stimuli consisted of pure sine wave tones with a duration of 100 ms and a random inter-stimuli interval of 800–1,100 ms. We presented 500 tones with a bimodally distributed pitch ranging from 400 to 800 Hz. We created the distribution as described below to avoid perceptually salient frequency shifts that could be used to categorize the two modalities. We first created two modes by shuffling 100,000 tones that were randomly drawn from two normal distributions with 50,000 tons each [*N* (500 Hz, 40 Hz) and *N* (700 Hz, 40 Hz)]. We then applied an iterative procedure that removed tones where the difference in Hz to the next tone in the sequence exceeded 80% of the maximum difference between all tones in the sequence. This process was repeated 1,000 times and resulted in a set of tones with a bimodally distributed pitch (Figure 1) without large frequency shifts between two trials. The first 500 tones in the set were used in the experiment. This procedure resulted in a stimulus distribution with range 405–802 Hz and with the left mode at 532 Hz, the center minima at 598 Hz, and the right mode at 677 Hz.^3^

#### EEG Analysis

In Experiment 2, we used the same approach to analyze the EEG data as in Experiments 1A and 1B, except that the MMR was computed as the difference between the average ERP of the tail intervals (deviant trials) and the mode intervals (frequent trials). At this stage of analysis, the center minima trials were not used; they were only used in the statistical analysis.

### Results and Discussion

We investigated our prediction using polynomial regression with the average negative normalized NLR as the dependent variable and interval (left tail/left mode/center minima/right mode/right tail) as the independent variable. We fitted both a second-order (i.e., with a linear and a quadratic term) and a fourth-order (i.e., with a linear, a quadratic, a cubic, and a quartic term) model (see Vassena et al., 2020 for a similar approach of using polynomial regression to evaluate the functional form of a brain response). The fourth-order model provided a better fit than the second-order model (AIC: −109.9 vs. −99.2). Furthermore, in the fourth-order model, both the quartic, *b* = −0.51, 95% CI = [−0.77, −0.24], *t*(90) = 3.9, *p* < 0.001, and the quadratic, *b* = −0.81, 95% CI = [−1.1, −0.55], *t*(90) = 6.2, *p* < 0.001, terms were significant, while the linear and cubic terms were not, indicating a shape with two peaks (see Figure 1, bottom right for an illustration). To ascertain that this finding was not the results of our choice to divide the data into five intervals, we ran the same models on all data without bins, with the average negative normalized NLR as the dependent variable and frequency as the independent variable. This analysis again showed a better fit for the fourth- than the second-order model (AIC: 10707 vs. 10712) with both significant quartic (*p* = 0.003) and cubic (*p* < 0.001) terms in the fourth-order model. Importantly, when fitting the same models to the data from the reference set in Experiments 1 (1A and 1B combined), the second-order model provided a better fit than the fourth-order model (BIC: 788.9 vs. 799.4). In the second-order model the quadratic term, *b* = −4.37, 95% CI = [−7.5, −1.2], *t*(202) = 2.76, *p* = 0.006, was significant. Taken together, these findings show that not only does the brain build probability distributions, it can do so flexibly for different underlying distributions, and it uses likelihood as the metric on which it builds the representation.

## General Discussion

One of the central questions in cognitive science is how the mind can parse massive amounts of unstructured data to navigate and learn about the physical and social environment (Tenenbaum et al., 2011; Conway, 2020). In the current study, we used an auditory learning paradigm to provide neural evidence that the brain can solve this problem by building probability distributions, even when no explicit instructions to sort, categorize, or learn the material are provided. We reasoned that three pieces of neural evidence were required for concluding that the brain builds representations of probability distributions; indexing, correspondence, and flexibility. In Experiment 1A, our results showed that the NLR indexed the individual items’ likelihood in a presented distribution. As the likelihood decreased, the brain response increased. Notably, this increase in brain response with decreased likelihood occurred even when keeping the presentation frequency of the tones constant. These results are in line with previous studies indicating that the amplitude of the MMN can capture the degree of deviance from the standard tone (Sams et al., 1985; Frost et al., 2016). However, we extend such findings by showing a brain response that captures a presented item’s likelihood.

Furthermore, Experiment 1A provided evidence for a correspondence between the distribution of the presented data and the NLR. By comparing the fit of the data to a normal curve to the fit of the data to a square curve, Experiment 1A also ruled out the possibility that the brain treated the data in the references and tail distributions as belonging to two different categories. Because we devised a new analysis method, we conducted Experiment 1B in an effort to replicate the findings from Experiment 1A. The results of Experiment 1B closely mimicked those of Experiment 1A. We replicated the correspondence analysis and one of the analyses of indexing. The results of the second analysis of indexing revealed the same pattern of results, but did not reach conventional levels of statistical significance.

Experiment 2 ruled out another alternative account of our findings, namely that the brain uses similarity rather than likelihood as the metric for building a representation of the data. More importantly, however, Experiment 2 provided the crucial evidence for flexibility that is necessary for concluding that the representation is sensitivity to the environment (Knill and Pouget, 2004; Ma et al., 2006; Fiser et al., 2010; Conway, 2020). That the NLR exhibited a functional form that mimics a bimodal distribution in Experiment 2 shows that the brain can flexibly build probability distributions for different underlying distributions of experienced data. Taken together, the findings in the two experiments provide neurophysiological evidence that the adult human brain can build a representation of the complex, global pattern of a probability distribution.

There are, of course, limitations to the present study. We suggest that evidence of a correspondence between the functional form of the NLR and the distribution of the presented data was needed to conclude that the brain can build a representation of a probability distribution. However, the mind could be probabilistic without exhibiting correspondence if, for example, it uses mental shortcuts that correspond to factual probabilities to make predictions about new data. Our data do, however, indicate the NLR exhibit correspondence. Furthermore, we do not find support for the possibility that the brain uses similarity as the metric on which it builds the representation or treats the data in the reference and tail distributions as belonging to different categories, both of which would be possible heuristics. There is thus little support for the idea that the brain is using a heuristic on the neural level to represent probabilities in the current study. However, future research should further investigate the intriguing possibility that the brain might rely on other rules-of-thumb than similarity to represent probabilities.

There are also methodological limitations that should be addressed. For example, the NLR measure is new, and although the metric seems to capture the structure of the stimuli, a more in-depth evaluation of the measure is warranted. When constructing the ROI, we relied on findings from the previous literature and visual inspection. A future evaluation of the NLR should better map the signal’s topographical distribution and its neural sources. Although the channel wise analyses provided in Supplementary Figures 4, 5, 7 show that the response is most clear over the scalp’s frontal and central areas, the exact neural origin is still unknown. Here, we mainly focused on the functional response on theoretical grounds to establish the phenomenon and pave the way for studies using MRI for reliable anatomical source localization. Further, the NLR’s topographical distribution overlaps with the MMR topography, but the relation between the MMR and the NLR is not clear. Although the NLR was based on the MMR and constructed as a single-trial equivalent to the MMR, they are not the same, and dedicated studies should disentangle the two.

Another methodological concern is that our trial rejection criteria of ranges >100 μV did not eliminate eye blinks sufficiently. However, there are two reasons why we do not think that eye blinks influenced our results. First, if eye blinks were unrelated to the stimuli, they would be evenly distributed across trials and cancel each other out over the hundreds of trials we presented. Secondly, if eye blinks were systematically related to the stimuli as startle responses, we would expect a bias for high or low pitch frequencies or the center frequencies seen in the bimodal experiment, but this is not seen in the data.

Furthermore, it is possible that the preprocessing of the data (i.e., band-pass filtering at 0.3–30 Hz and resampling at 100 Hz) introduced confounding effects, in line with Tanner et al. (2015). To evaluate the influence of preprocessing parameters, we reanalyzed both Experiment 1 (A and B) and Experiment 2 without downsampling (using the recorded 1,000 Hz) and changing the band-pass to 0.1–30 Hz. The analysis did not change our interpretation of the data but instead support the results’ robustness (see Supplementary Figures 3, 6). Independent replications, which we encourage, could further strengthen these conclusions.

An essential part of probabilistic accounts of cognition and perception is that existing knowledge is updated in the light of new data (Knill and Pouget, 2004; Ma et al., 2006; Fiser et al., 2010; Tenenbaum et al., 2011; Winkler and Czigler, 2012; Clark, 2013; Pouget et al., 2013; Ma and Jazayeri, 2014; Conway, 2020). Our results provide evidence for the biological plausibility of an essential tenant of these accounts; that the brain represents probability distributions. The design of the current study did not allow us to evaluate how the representation evolves over time. However, the finding that the brain can build probability distributions flexibly for different underlying distributions in the data suggests that the representation is learned and fine-tuned as new information become available. One special case of a probabilistic account is the Bayesian account, which proposes that internal representations of probability distributions are updated in the presence of new data by applying Bayes’ rule (Tenenbaum et al., 2011; Ma and Jazayeri, 2014). It will be an exciting venture for future research to investigate how the brain updates the representation of probability distributions and if it does so by using Bayes’ rule or some other updating rule.

Humans learn about the world in several ways, by active exploration and through social and cultural transmission. Our findings pave the way for a new, neurologically grounded method of investigating learning with direct implications for developmental and cognitive psychology, as well as clinical research and research in judgment and decision-making. Questions that now become directly accessible include the ontology (Tenenbaum et al., 2011; Conway, 2020) of information processing and the conditions under which data are encoded as continuous probability distributions versus discrete categories. A critical question for future research will be to investigate if a neural representation of probability distributions is present already in infancy such that it can be a foundation for learning about the world (Tenenbaum et al., 2011; Conway, 2020). Previous work using similar paradigms as in the current study has shown that infants can learn statistical regularities in the environment (Saffran et al., 1996; Fiser and Aslin, 2002; Kirkham et al., 2002; Xu and Garcia, 2008). However, these studies have focused on local probabilistic features (Dehaene et al., 2015) of the presented data, such as transitional probabilities (Saffran et al., 1996), and there is yet little evidence, both on a behavioral and a neural level, for the infant mind being able to represent the complex, global patterns of a probability distribution.

We conclude by noting that the NRL is a novel tool with the potential to significantly enhance our understanding of the probabilistic nature of the human mind and its ability to learn and re-organize as a function of experience.

## Data Availability Statement

The datasets generated for this study can be found in online repositories. The names of the repository/repositories and accession number(s) can be found below: https://osf.io/hg5ps/.

## Ethics Statement

The studies involving human participants were reviewed and approved by Regionala Etikprövningsnämnden. The patients/participants provided their written informed consent to participate in this study.

## Author Contributions

The authors worked as a team, and their individual contributions were equally important to the completion of the project. ML and GG conceived the studies. ML, PN, and GG designed and planned the studies. GG and PN supervised the data collection. PN devised the analysis method and analyzed the EEG data. ML and GG wrote the first draft. All authors contributed to revising the first draft. All authors approved the submitted version of the manuscript.

## Conflict of Interest

The authors declare that the research was conducted in the absence of any commercial or financial relationships that could be construed as a potential conflict of interest.

## Footnotes

1 Degrees of freedom for the fixed effects of all linear mixed models were determined using Satterthwaite’s method.

2 The negative normalized NLR (nnNLR) for the *i*:th tone the *k*:th participant was calculated as nnNLR_i,k_ = −(NLR_i,k_ − μ_k_)/σ_k_.

3 We established the parameters of the distributuon by fitting a probability distribution to the frequencies of all trials, using the “fitdist” command in Matlab with a normal kernel.
