# Supplementary material for: Can the Brain Build Probability Distributions?
Source: Front Psychol. 2021 Mar 25;12:596231. doi: 10.3389/fpsyg.2021.596231 (PMC8026893; doi:10.3389/fpsyg.2021.596231)
Supplement: Supplementary file 1 [file Data_Sheet_1.docx]

Supplementary Information for

The Brain Builds Probability Distributions

Marcus Lindskog^1,2 *^, Pär Nyström^1^, & Gustaf Gredebäck^1^

Correspondence to: [marcus.lindskog@psyk.uu.se](mailto:xxxxx@xxxx.xxx)

# Supplementary Methods


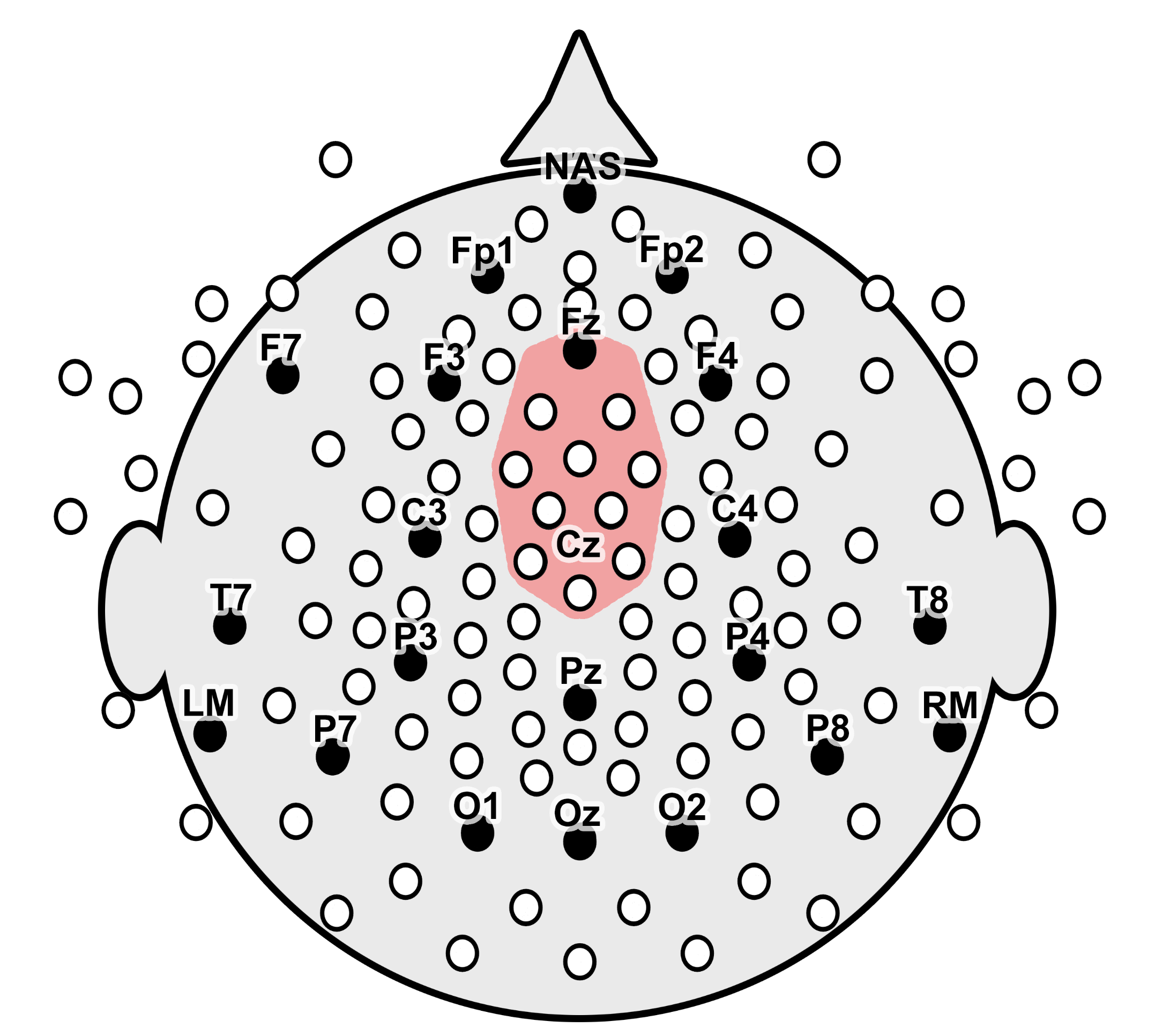


*Figure S1.* ROI channel locations are marked using the red area, with 10-20 standard channel locations superimposed.

# Slow frequency noise and its effect on single-trial variability

Figure S2 summarizes a simulation demonstrating that a difference measure within single trials reduces variability compared to using absolute peak values.

***
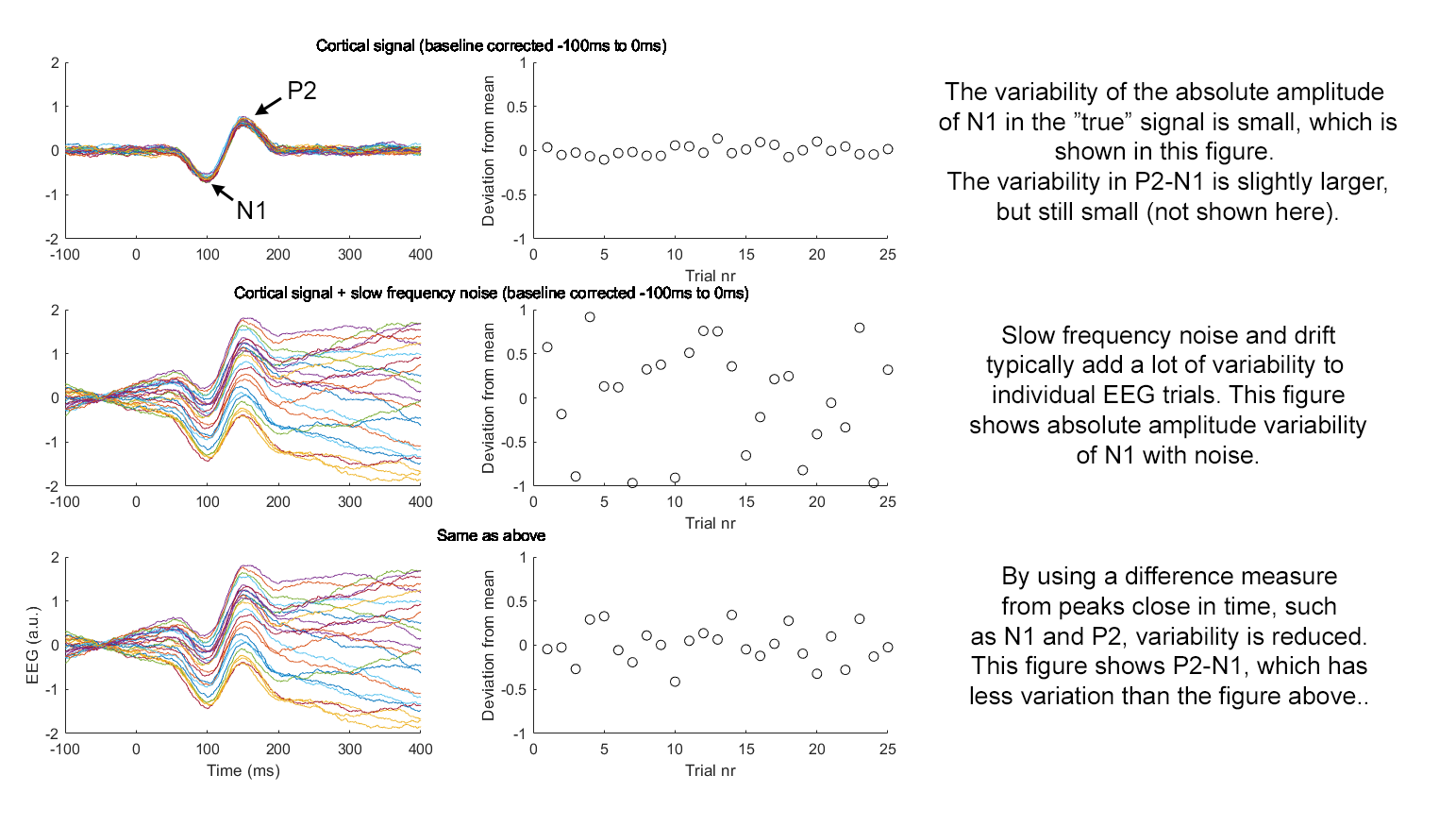
***

*Figure S2.* Stimulation of slow frequency noise and its effect on single-trial variability.

# Supplementary Results

# Experiment 1A and 1B

## Robustness check

We replicated the findings illustrated in Figure 3 (bottom left) of the main text, using different EEG preprocessing parameters to assess parameter robustness. The data was bandpass filtered at 0.1-30Hz, instead of 0.3-30Hz, and there was no resampling from 1000Hz to 100Hz. The results, illustrated in Figure S3, are conceptually similar and did not change our interpretation of the main results.
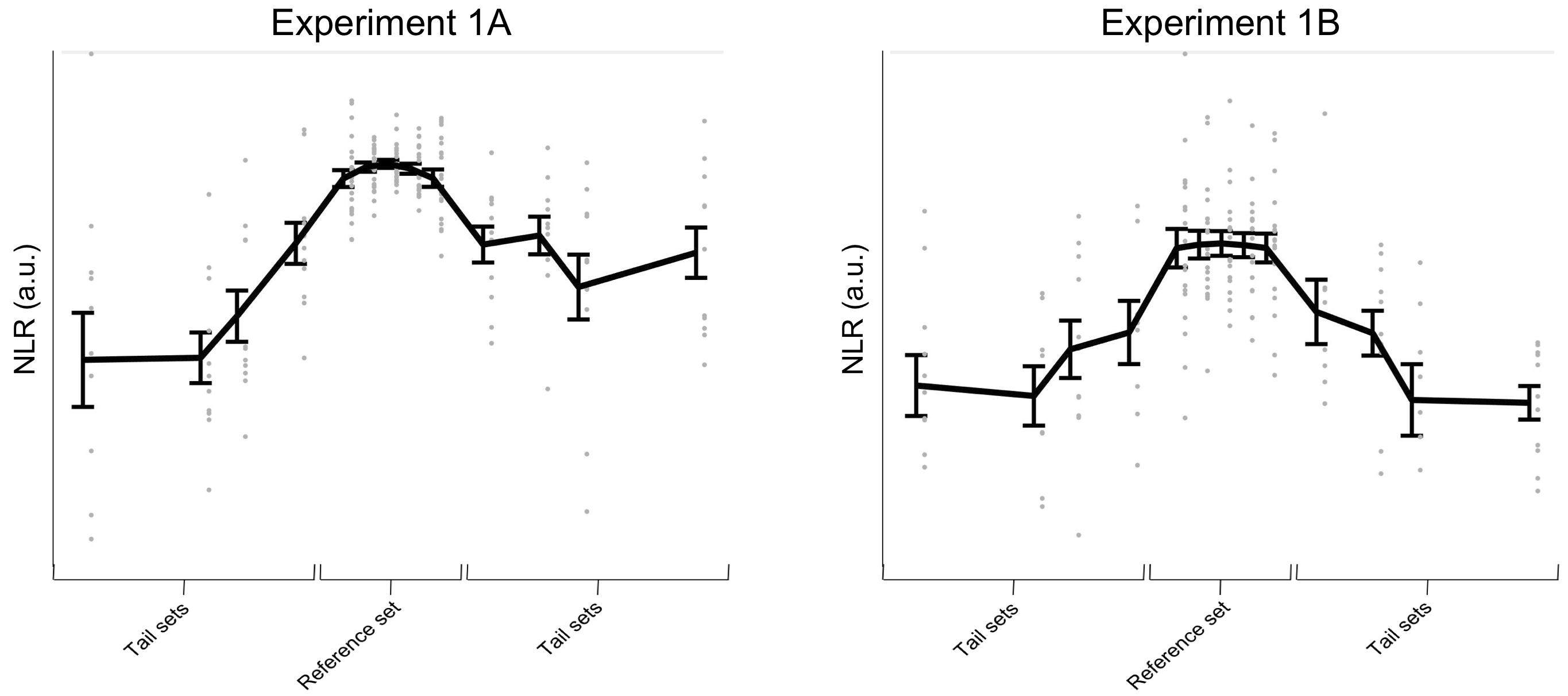


*Figure S3.* Individual data points from Experiments 1A and 1B with the mean and standard error (whiskers). The figure depicts the negative normalized NLR. In contrast to Figure 3 of the main text, the figure shows data that were bandpass filtered at 0.1-30Hz instead of 0.3-30Hz, and there was no resampling from 1000Hz to 100Hz.

## Topographical analyses


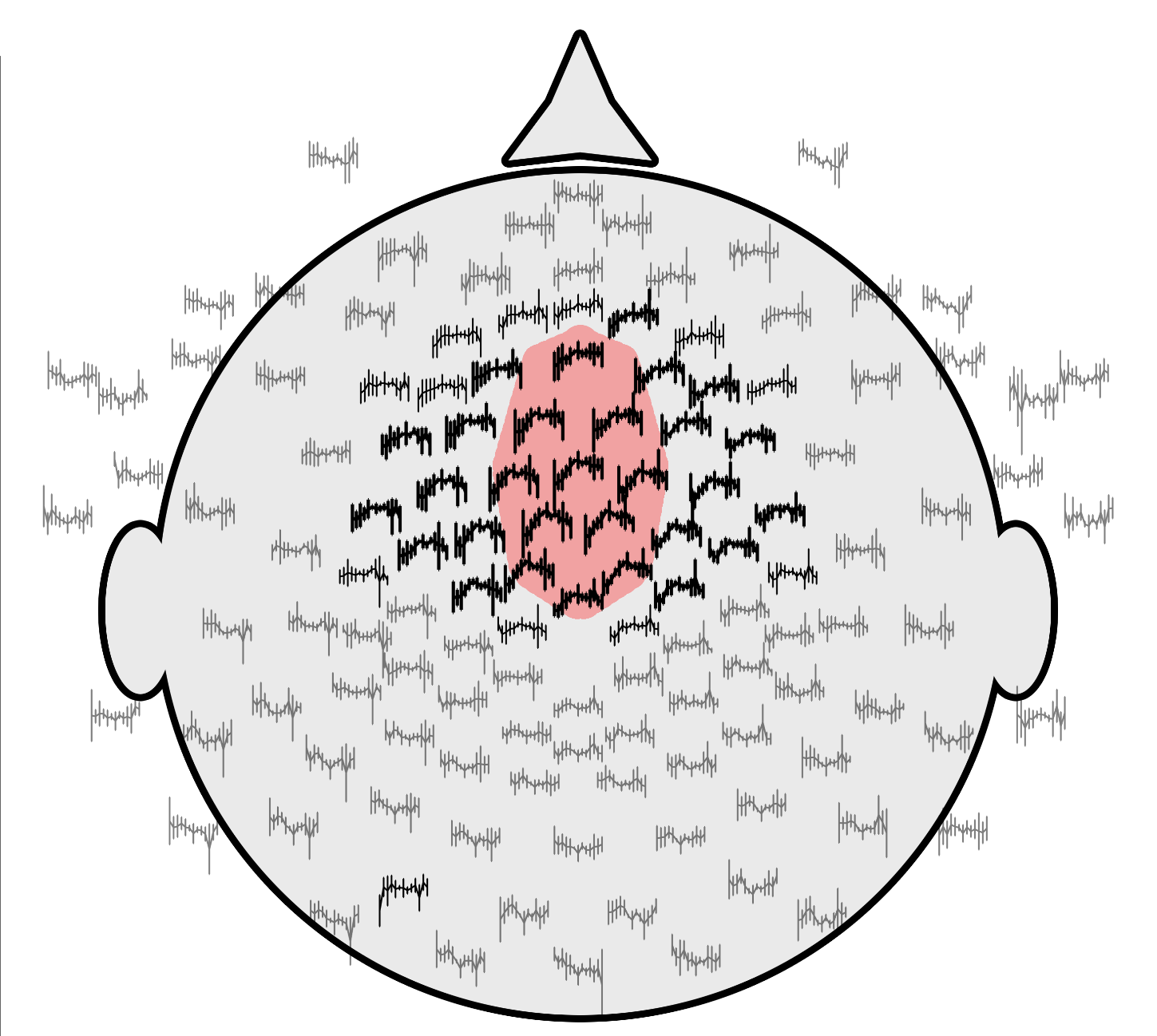


*Figure S4.* Topographic plot of the NLR from Experiment 1A, where data is binned and normalized as in main Figure 3 (bottom) for each EEG channel. Whiskers show 95% confidence intervals. Linear mixed models on the form NLR ~ Abs(z) + (1|subject) were performed for each channel. Channels with significant negative NLR slopes over absolute Z-score are plotted in black, and non-significant effects in grey. Channels that were significant after Bonferroni correction for 128 analyses are marked with bold black lines. The ROI used in the main text is marked with a red patch


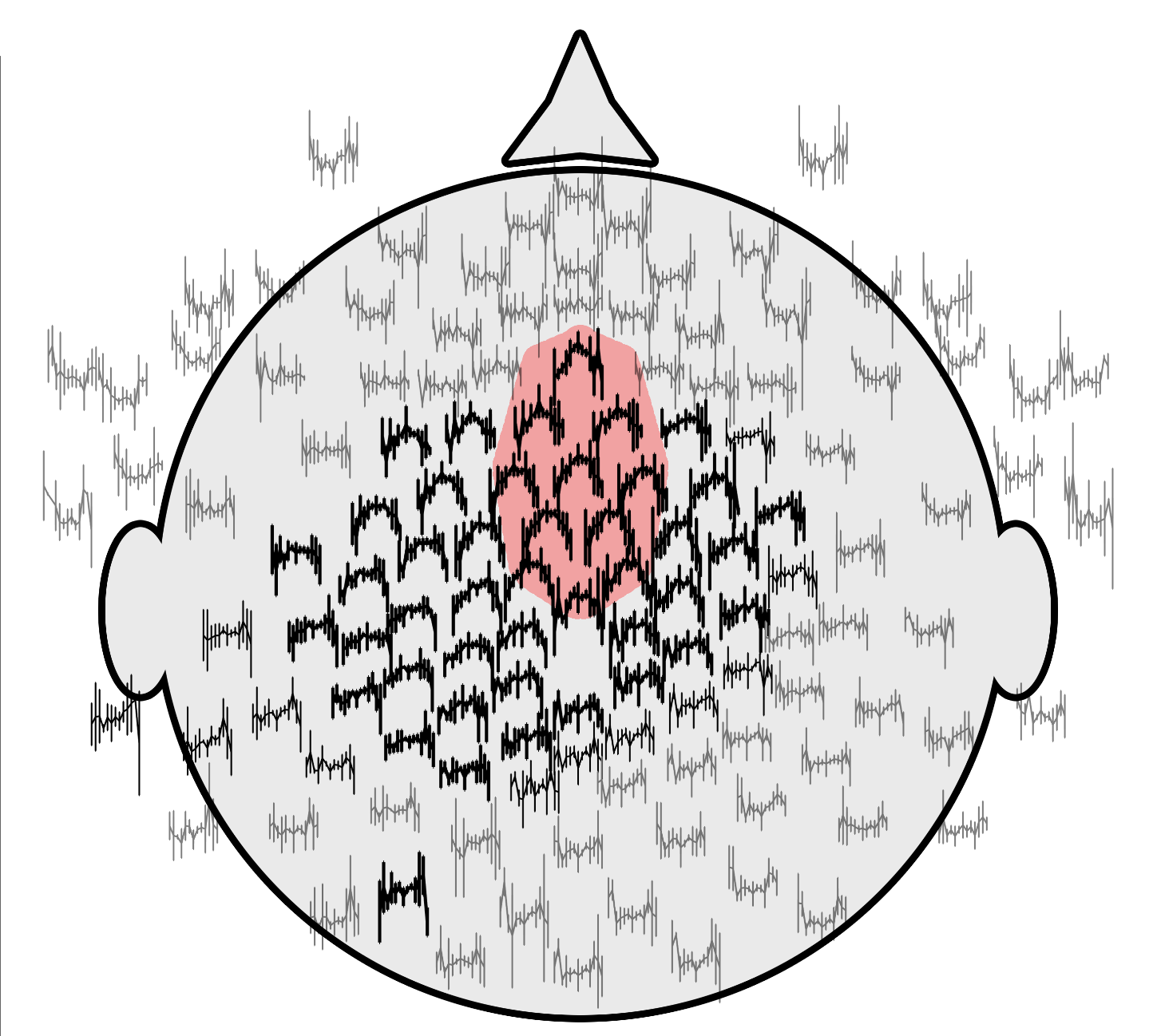


*Figure S5.* Topographic plot of the NLR from Experiment 1B, where data is binned and normalized as in main Figure 3 (bottom left) for each EEG channel. Whiskers show 95% confidence intervals. Linear mixed models on the form NLR ~ Abs(z) + (1|subject) were performed for each channel. Channels with significant negative NLR slopes over absolute Z-score are plotted in black, and non-significant effects in grey. Channels that were significant after Bonferroni correction for 128 analyses are marked with bold black lines. The ROI used in the main text is marked with a red patch

# Experiment 2

## Robustness check

We replicated the findings illustrated in Figure 3 (bottom right) of the main text, using different EEG preprocessing parameters to assess parameter robustness. The data was bandpass filtered at 0.1-30Hz, instead of 0.3-30Hz, and there was no resampling from 1000Hz to 100Hz. The results, illustrated in Figure S6, are conceptually similar and did not change our interpretation of the main results.


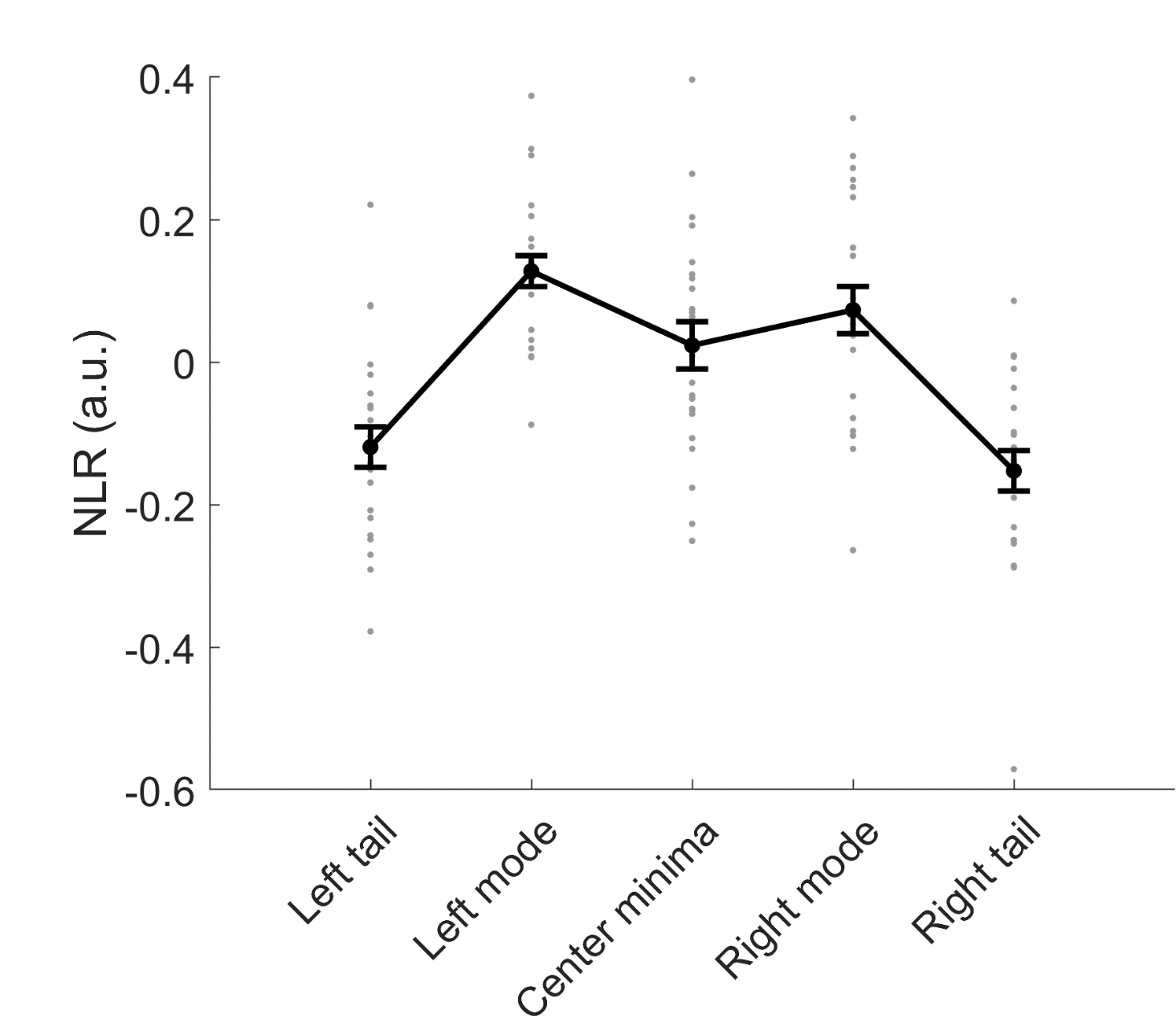


*Figure S6.* Individual data points from Experiment 2 with the mean and standard error (whiskers). The figure depicts the negative normalized NLR. In contrast to Figure 3 of the main text, the figure shows data that were bandpass filtered at 0.1-30Hz instead of 0.3-30Hz, and there was no resampling from 1000Hz to 100Hz.

## Topographical analyses


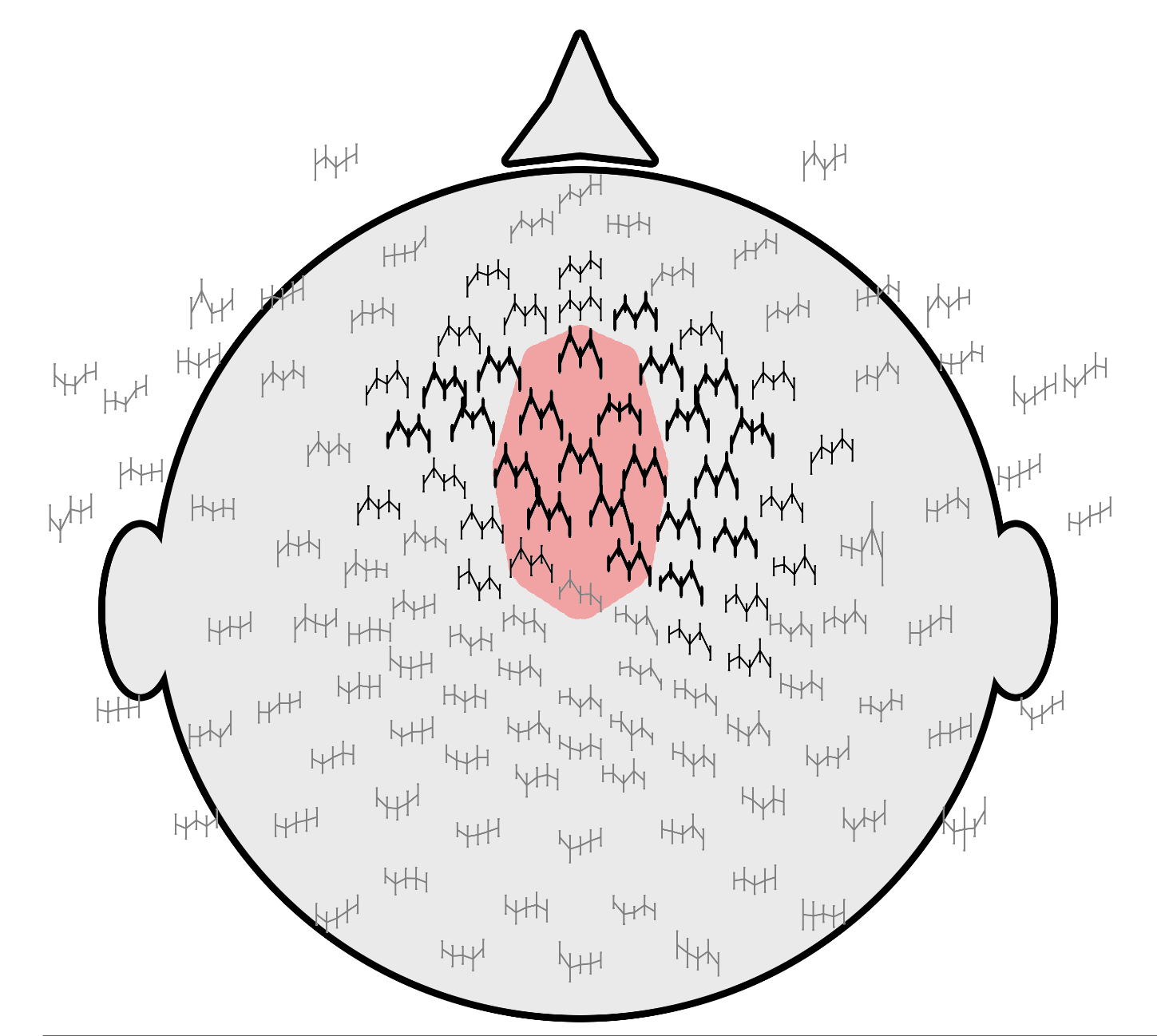


*Figure S7.* Topographic plot of the NLR in Experiment 2 for each EEG channel. As in main Figure 3 (bottom right), 50 trial intervals were used for the analysis, coming from the left tail, left mode, center minima, right mode and right tail of the stimuli distribution, respectively. Linear mixed models on the form NLR ~ condition + (1|subject) was performed for each channel, where condition was a fixed factor with three levels: tail / mode / center. Channels with significant effects of condition are plotted in black, and non-significant effects in grey. Channels that were significant after Bonferroni correction for 128 analyses are marked with bold black lines. The ROI used in the main text is marked with a red patch.
